# Ceramide Metabolic Pathways and Their Regulation in Adolescent Acne Vulgaris

**DOI:** 10.3390/ijms27198737

**Published:** 2026-09-30

**Authors:** Yvwei Gou, Hua Wu, Xingyi Wu, Guolong Dong, Haodong Li, Huanxiang Yuan, Li Li

**Affiliations:** Beijing Key Laboratory of Plant Resource Research and Development, Beijing Technology and Business University, Fucheng Road, Haidian District, Beijing 100048, China; yuwei_gou@163.com (Y.G.);

**Keywords:** ceramide, acne vulgaris, skin barrier, ceramide synthesis pathways, PPARα, ultra-long-chain fatty acid

## Abstract

Ceramides constitute approximately 50% of the lipid mass in the stratum corneum and are essential for epidermal permeability barrier function. In acne vulgaris, barrier disruption is accompanied by altered ceramide composition, yet the molecular mechanisms that connect acne-associated signals to specific changes in ceramide metabolism remain incompletely understood. This review integrates evidence from lipidomic profiling, enzymatic pathway characterization, and inflammatory signaling studies to examine how ceramide biosynthesis and turnover are modulated in acne-prone skin. Particular attention is directed to the elongation of very-long-chain fatty acids (VLCFAs) and their incorporation into ceramides by ceramide synthase 3 (CerS3). In cultured keratinocytes, *Cutibacterium acnes* (*C. acnes*)-derived short-chain fatty acids (SCFAs) have been reported to suppress CerS3 and 3-ketodihydrosphingosine reductase (KDSR) through peroxisome proliferator-activated receptor α (PPARα), limiting ultra-long-chain fatty acid (ULCFA) supply. We propose a working model in which SCFAs act via PPARα to upregulate ELOVL-family elongases while suppressing CerS3 and KDSR, reducing ULCFA-ceramides and compromising barrier integrity. The CerS3–KDSR node is a candidate regulatory point coupling microbial metabolites to barrier impairment and inflammation. However, this model rests largely on in vitro evidence and requires in vivo validation. Clarifying these regulatory relationships may inform the development of barrier-restorative strategies as adjuncts to conventional acne therapy.

## 1. Introduction

Acne vulgaris is a chronic inflammatory condition of the pilosebaceous unit, affecting approximately 85% of adolescents and young adults worldwide [1,2]. Lesions predominate on the face, chest, and back, regions with the highest density of sebaceous glands. The disorder arises from the interplay of four pathogenic processes: sebaceous gland hyperplasia with excessive sebum production, altered follicular keratinization, colonization by *Cutibacterium acnes* (*C. acnes*), and innate and adaptive immune activation [1,2]. While adolescent acne shows a slight male predominance, adult acne is more prevalent in women, reflecting the influence of hormonal profiles, cosmetic use, medications, and chronic psychosocial stress on disease persistence [3].

Among the pathogenic drivers, epidermal barrier dysfunction has received increasing attention. Acne-affected skin exhibits elevated transepidermal water loss (TEWL) and reduced stratum corneum hydration, both indicative of a compromised barrier [4]. These functional deficits correlate with altered lipid composition: total ceramide levels are reported to be reduced in stratum corneum analyses when normalized to protein content [4,5], whereas total free fatty acid (FFA) levels remain relatively unchanged [4]. Subclass- and chain-length-resolved profiling more consistently reveals a redistribution of ceramides toward shorter acyl-chain species with depletion of long- and ultra-long-chain subclasses (Section 3.1). Ceramide imbalance may independently contribute to inflammation and lesion progression, as ceramides and their metabolites serve dual roles, structural components of the epidermal permeability barrier and bioactive signaling molecules that modulate inflammatory pathways [5].

Recent lipidomic and transcriptomic studies have begun to map the enzymatic machinery responsible for ceramide dysregulation in acne [6,7]. However, the literature remains fragmented: compositional studies document what changes in ceramide profiles, while mechanistic studies on synthetic enzymes rarely connect their findings back to the compositional data. This review integrates these two bodies of evidence, ceramide lipidomics and pathway enzymology, to identify the regulatory nodes at which acne-associated signals converge on ceramide metabolism. We focus explicitly on the *de novo* synthesis pathway and fatty acid elongation, the two processes whose enzymatic steps determine the chain-length profile of epidermal ceramides, and examine their transcriptional modulation by acne-associated microbial metabolites and inflammatory mediators.

## 2. Literature Search Strategy

A narrative literature search was conducted in PubMed/MEDLINE, Web of Science Core Collection, and Scopus. The search covered records from database inception through 31 December 2025.

The search strategy combined terms related to acne (“acne vulgaris” OR “acne” OR “adolescent acne”), epidermal lipids and barrier function (“ceramide” OR “sphingolipid” OR “stratum corneum” OR “skin barrier” OR “skin surface lipid” OR “epidermal lipid”), and ceramide metabolic pathways or regulatory mechanisms (“ceramide synthase 3” OR CerS3 OR KDSR OR ELOVL OR DES1 OR DES2 OR PNPLA1 OR “acid sphingomyelinase” OR β-glucocerebrosidase OR *Cutibacterium acnes* OR PPARα OR inflammation). These concepts were combined using Boolean operators as appropriate for each database.

Priority was given to human studies of lipid composition, ceramide profiles, barrier function, or circulating sphingolipids in acne, and to experimental studies on ceramide biosynthesis, fatty acid elongation, or epidermal lipid regulation. Selected studies in atopic dermatitis and psoriasis were included only when directly relevant to conserved epidermal ceramide pathways and were distinguished from acne-specific evidence. Reference lists of key articles were screened. Studies unrelated to epidermal ceramide or lipid biology were excluded.

## 3. Differences in Epidermal Lipid Composition Between Adolescent Acne Patients and Healthy Individuals

The extracellular spaces of the stratum corneum are filled with multilamellar lipid structures composed predominantly of ceramides (~50%), cholesterol (~27%) and its esters (~10%), cholesterol sulfate (2–5%), and FFAs (10–15%) [8]. These lipids are delivered by lamellar body exocytosis at the stratum granulosum–stratum corneum interface and organized into tightly packed, repeating bilayers that constitute the epidermal permeability barrier. Comparative lipidomic analyses have identified consistent differences in the abundance and chain-length distribution of these lipid classes between acne-affected and healthy skin.

### 3.1. Ceramide Composition and Chain-Length Changes in Acne

The barrier function of the stratum corneum is classically described by the brick-and-mortar model, in which terminally differentiated corneocytes (bricks) are embedded in a continuous matrix of intercellular lipids (mortar) [9,10]. Ceramides constitute the quantitatively dominant and structurally important component of this lipid mortar.

Ceramides are sphingolipids formed by N-acylation of a sphingoid base with a fatty acid (FA). In human stratum corneum, over 300 species have been identified, with diversity arising from both the sphingoid base and the N-acyl FA chain [11,12].

**Figure 1:**
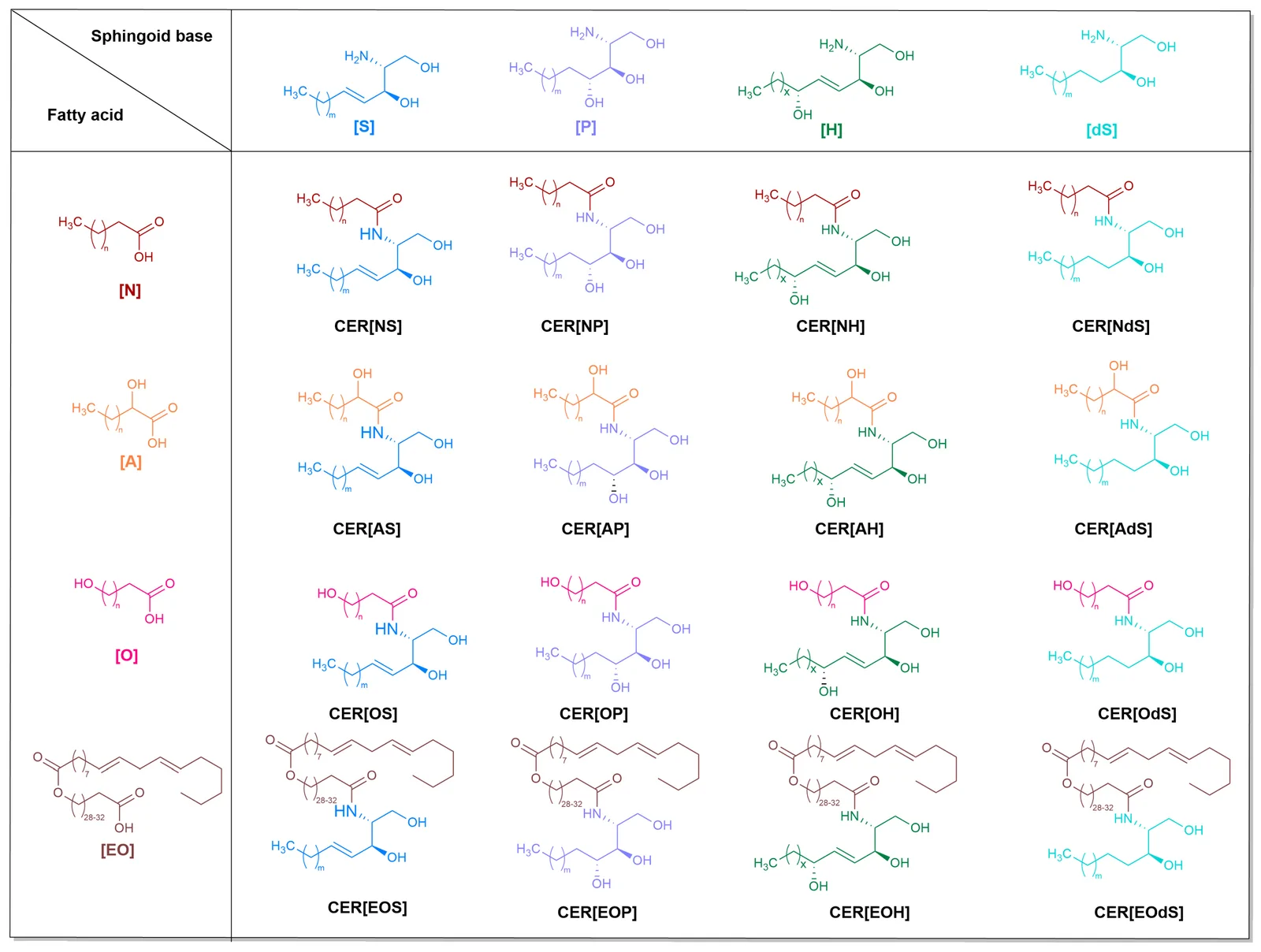
Chemical structures of different ceramide subtypes.

The widely used ceramide nomenclature classifies these lipids by the combination of their FA and sphingoid base moieties (Table 1, Figure 1) [13,14,15]. The first letter designates the amide-linked FA: N (non-hydroxy), A (α-hydroxy), O (ω-hydroxy), and EO (esterified ω-hydroxy). The last letter designates the sphingoid base: S (sphingosine), P (phytosphingosine), H (6-hydroxysphingosine), and dS (dihydrosphingosine). This yields up to 16 subclasses [13,14,15].

Among these, the ω-hydroxy ceramides, CER[EOS], CER[EOP], and CER[EOH], serve a specialized function. After synthesis, these ultra-long-chain species (FA chain length ≥ C28) are covalently attached to the outer surface of corneocytes via transglutaminase-mediated ester linkages, forming the corneocyte lipid envelope (CLE). The CLE provides a hydrophobic scaffold onto which the intercellular lipid lamellae are organized, thereby serving as the architectural foundation of the epidermal barrier [16].

Acne-affected skin exhibits elevated TEWL and reduced stratum corneum hydration, functional deficits that correlate with decreased total ceramide levels. Pappas et al. conducted a direct analysis of the stratum corneum ceramide profile in acne, examining stratum corneum ceramides normalized to protein, an approach that minimizes confounding by surface sebum dilution, in seven adolescent male acne patients aged 13–18 years and ten healthy controls [5]. Across 283 individual ceramide species spanning 10 subclasses, they found overall lower ceramide abundance in acne-affected skin, with significant reductions in CER[EOH], CER[EOS], CER[AH], and CER[NH]. CER[EOS] and CER[EOH] are essential for stratum corneum organization and barrier function; their depletion is expected to compromise lamellar architecture. CER[NH] and CER[AH], as critical subclasses that participate in highly ordered lamellar lipid structures, directly modulate TEWL and maintain stratum corneum hydration [5]. In acne-affected skin, the depleted CER[NH], CER[AH], and CER[EOH] species were predominantly those bearing H18 sphingoid bases conjugated to non-hydroxylated (N24/N26) and α-hydroxylated (A24/A26) acyl chains [5]. Similar CER[NH] and CER[EOH] reductions occur in atopic dermatitis (AD) [17].

The acyl chain composition of the depleted ceramides points to a specific biosynthetic vulnerability. The enrichment of C24 and longer acyl chains in the affected subclasses implicates the FA elongation machinery and the ceramide synthase (CerS) isoforms responsible for incorporating very-long-chain FAs (VLCFAs) and ultra-long-chain FAs (ULCFAs) into ceramides, enzymes whose expression is directly modulated by acne-associated stimuli (Section 4.3).

Serum sphingolipid profiling provides complementary evidence. Kaya et al. reported significantly elevated serum C16 sphingomyelin (SM) and ceramide-1-phosphate (C1P) in acne patients, alongside significantly reduced very-long-chain C24 ceramide [18]. The elevation of short-chain sphingolipid species in circulation, coupled with depletion of long-chain ceramides in the stratum corneum, suggests a systemic shift in sphingolipid metabolism affecting both cutaneous and circulating compartments. Su et al. analyzed facial skin surface lipids in young women with acne and found elevated unsaturated FAs and shorter average ceramide chain lengths compared to healthy controls [19]. This pattern mirrors the findings in male patients, suggesting that the direction of chain-length shortening is consistent across the sexes; whether sex-specific hormonal effects further modulate ceramide composition remains to be determined (Section 3.3).

It should be noted that not all studies report reduced total ceramides. Two recent quantitative lipidomic studies of skin surface lipids instead reported increased total ceramide abundance in acne: Cheng et al. in adults (51 patients versus 38 controls) [20] and Tan et al. in moderate-to-severe acne (21 versus 20), where the elevation was significant only in female patients (*p* = 0.009) [21]. These apparent discrepancies are most readily explained by differences in the lipid pool measured: surface-lipid (sebum) analyses are confounded by sebum dilution and measure absolute abundance without protein normalization, whereas stratum corneum ceramide profiling normalized to protein avoids this confound [5]. By contrast, the direction of chain-length shortening and depletion of ultra-long-chain subclasses is consistent across the studies that resolved chain length and subclasses [5,19,22].

This ceramide pattern overlaps with, but is not identical to, that of other inflammatory dermatoses. AD also shows reduced CER[NH] and CER[EOH] and accumulation of short-chain ceramides [15,17,23], whereas psoriasis is characterized by a selective reduction in CER[NP] [13]. Seborrheic dermatitis, another sebaceous-gland-associated inflammatory condition, is driven by the yeast *Malassezia* rather than *C. acnes* [24], indicating that microbial–lipid interactions are pathogen-specific. Because ceramides are essential for stratum corneum barrier function, the ceramide depletion and ULCFA shortening observed in acne would be expected to impair barrier integrity and may thereby increase susceptibility to opportunistic fungal colonization. Such fungi rarely breach an intact stratum corneum but can exploit barrier defects, consistent with a One Health perspective linking skin-barrier integrity to infection susceptibility [25].

Collectively, the compositional data define a conserved ceramide phenotype in acne: depletion of specific long-chain and ultra-long-chain ceramide subclasses (CER[NH], CER[AH], CER[EOH], CER[EOS]), a relative increase in shorter-chain species, and a systemic elevation of pro-inflammatory sphingolipid metabolites (C16 SM, C1P). The direction of this phenotype is consistent across sexes, although its magnitude may be subject to sex-hormonal modulation (Section 3.3).

### 3.2. Free Fatty Acids, Cholesterol, and Other Lipids

The non-ceramide lipid fraction of the stratum corneum, cholesterol (27%), cholesterol esters (10%), cholesterol sulfate (2–5%), and FFAs (10–15%), also contributes to barrier function [8]. Cholesterol maintains the fluidity and mechanical stability of the lipid lamellae [26]. The majority of barrier-relevant non-esterified fatty acids (NEFAs) and ceramide-bound FAs in the stratum corneum are saturated, unbranched, with hydrocarbon chain lengths from C16 to C26 [27]. FFAs contribute to barrier function, maintenance of the acidic surface pH (the acid mantle), and antimicrobial defense [28].

Lipidomic studies have documented altered FA composition in acne patients. Zhou et al. found that young male acne patients exhibit increased levels of total FAs and unsaturated NEFAs, elevated cholesterol, and decreased levels of long-chain saturated FAs, saturated NEFAs, linoleic acid, and average ceramide chain length [22]. A subsequent study in adolescent acne patients with darker skin tones replicated these findings: elevated unsaturated NEFAs, squalene, and triglycerides, with shorter average ceramide chain lengths [29]. The consistent reduction in average ceramide chain length across demographic groups further supports the hypothesis that the FA elongation pathway is a key regulatory node affected in acne (Section 3.1).

### 3.3. Hormonal Influences on Ceramide Composition in Acne

Hormonal signaling may shape the epidermal ceramide profile through at least three distinct, but potentially overlapping, routes, although no study has yet established a direct androgen-mediated effect on CerS3, KDSR, or ELOVL expression. First, lipid dilution: androgens stimulate sebum secretion, and increased sebum flow may dilute linoleic acid within the follicular microenvironment, limiting the supply of the essential ω-esterified linoleate on acylceramides. Supporting this, the esterified linoleate content of comedonal acylceramides falls from approximately 41% to 6% [30], and topical application of 2.5% linoleic acid reduces follicular microcomedone size by about 25% [31]. Second, secretory mechanics: testosterone delays permeability-barrier recovery after disruption, an effect attributed to impaired lamellar-body formation and secretion rather than to altered lipid synthesis [32]. Third, compositional shifts: stratum corneum sphingolipid composition varies with age and sex, with altered relative abundances of specific ceramide subclasses reported across adolescence into adulthood in females [33]. Androgen-dependent sebaceous lipid synthesis may itself require peroxisome proliferator-activated receptor (PPAR) signaling, since the conversion of testosterone to 5α-dihydrotestosterone and the associated synthesis of sebaceous lipids are regulated by the PPAR ligand linoleic acid in sebocytes [34]; whether this intersects the ceramide pathway is unknown. Oral isotretinoin, which profoundly alters androgen-driven sebaceous output, increases total comedonal ceramide content by approximately 19% after six weeks [35]. Together, these observations suggest that hormonal effects on ceramide composition are likely indirect—mediated through sebum output, lamellar-body secretion, and differentiation—and each of these hypotheses awaits direct in vivo validation in acne.

## 4. Ceramide Metabolic Pathways Relevant to Adolescent Acne

### 4.1. Overview of Ceramide Synthesis and the De Novo Pathway

**Figure 2:**
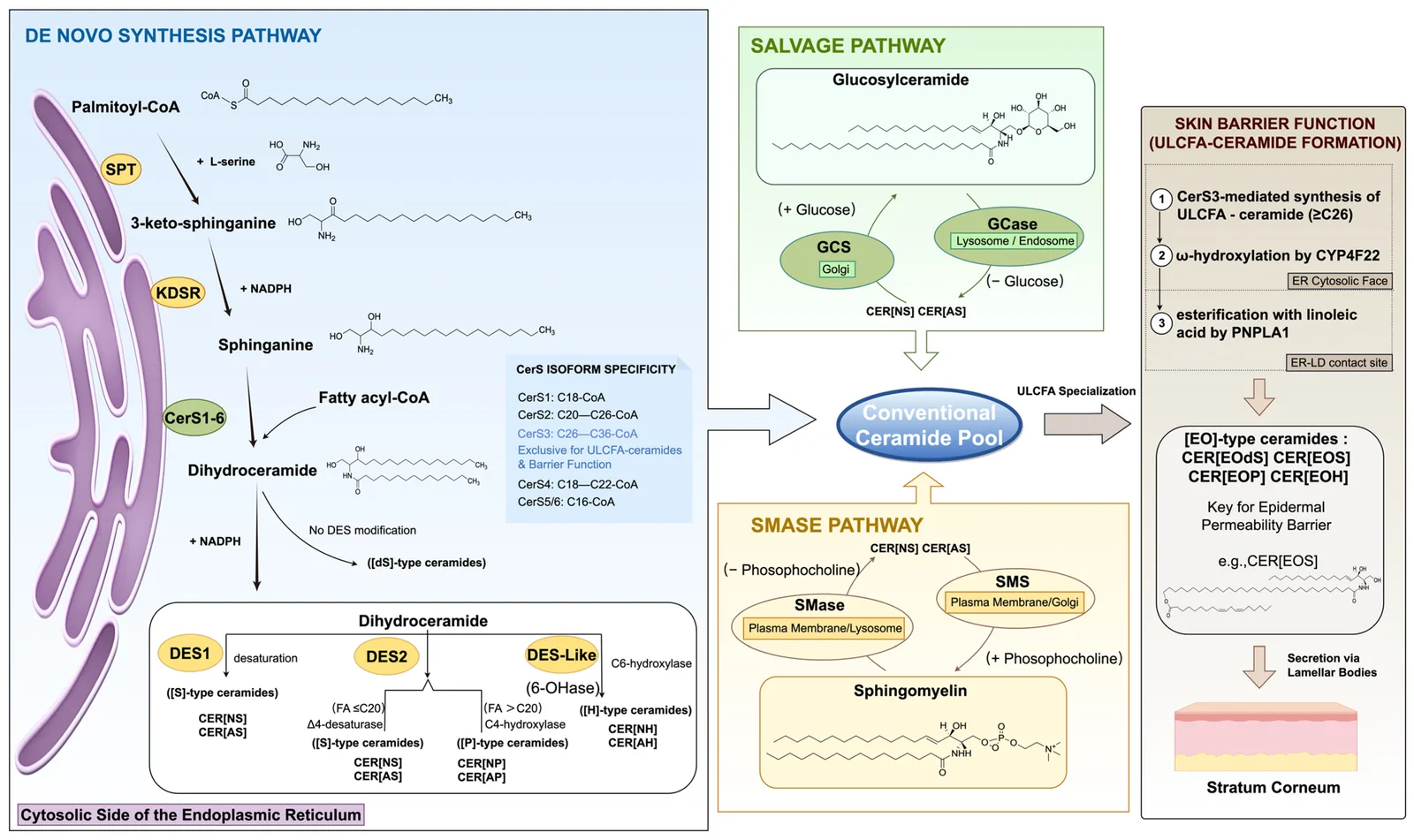
Three synthesis pathways of ceramides.

Epidermal ceramides are generated through three principal routes: *de novo* synthesis in the endoplasmic reticulum (ER), hydrolysis of sphingomyelin by acid sphingomyelinase (aSMase), and hydrolysis of glucosylceramide by β-glucocerebrosidase (Figure 2). The *de novo* pathway, which operates in keratinocytes of the stratum spinosum, proceeds through four sequential enzymatic steps.

Step 1: Serine palmitoyltransferase (SPT) condenses palmitoyl-CoA with L-serine to form 3-ketodihydrosphingosine [36]. As the rate-limiting enzyme, SPT governs the rate and total yield of ceramide synthesis.

Step 2: 3-Ketodihydrosphingosine reductase (KDSR). The nicotinamide adenine dinucleotide phosphate (NADPH)-dependent reduction of 3-ketodihydrosphingosine to dihydrosphingosine is catalyzed by KDSR [37]. This enzyme, located on the cytosolic face of the ER membrane, is critical for VLCFA-ceramide synthesis because the dihydrosphingosine it produces is the obligate substrate for CerS3, which is almost exclusively responsible for ULCFA-ceramide production.

Step 3: Ceramide synthase (CerS). Dihydrosphingosine is N-acylated with a fatty acyl-CoA by one of six CerS isoforms (CerS1–6) to yield dihydroceramide, with each isoform exhibiting distinct acyl-chain-length specificity that determines the tissue ceramide profile [38,39]. Among them, CerS3 is the sole isoform capable of incorporating ultra-long-chain fatty acids (ULCFAs, ≥C26) into ceramides and is therefore essential for epidermal barrier function. CerS3-deficient mice show a complete loss of epidermal ceramides with acyl chains ≥ C26 and a lethal barrier defect [40], and biallelic CerS3 mutations cause autosomal recessive congenital ichthyosis in humans [41]. CerS3 generates the ULCFA-ceramide backbone (e.g., CER[NS] with a C28–C36 acyl chain); subsequent ω-hydroxylation to produce ω-hydroxy ceramides (CER[OS], CER[EOS]) and α-hydroxylation to produce α-hydroxy ceramides (CER[AS]) are catalyzed by separate fatty acid hydroxylases (e.g., CYP4F22), not by CerS3 itself [40,42]. Other CerS isoforms use shorter acyl-CoAs (e.g., CerS1, C18; CerS2, C20–C26; CerS4, C18–C22; CerS5/6, C16) and contribute to the overall ceramide profile but cannot substitute for CerS3 in ULCFA-ceramide synthesis [39].

Step 4: Dihydroceramide desaturase (DES). The introduction of a 4,5-trans double bond into dihydroceramide to ceramide, yielding sphingosine-based ceramides (e.g., CER[NS], CER[AS], CER[EOS]) [43]. DES2 is bifunctional, acting as a Δ4-desaturase with C20 or shorter acyl chains to produce sphingosine-based ceramides and as a C4-hydroxylase with longer chains to produce phytosphingosine-based ceramides (e.g., CER[NP], CER[AP]). 6-Hydroxysphingosine-based ceramides (e.g., CER[NH], CER[AH]) are generated by a distinct C6-hydroxylase that remains to be fully characterized. The ω-esterified subclass (CER[EOS], CER[EOP], CER[EOH]) additionally requires acylation of the ω-hydroxyl group with linoleic acid by PNPLA1 [42].

Following *de novo* synthesis, ceramides are glycosylated to glucosylceramide or conjugated with phosphocholine to form SM in the Golgi apparatus. These derivatives are packaged into lamellar bodies and secreted into the intercellular space at the stratum granulosum–stratum corneum interface, where β-glucocerebrosidase and acid sphingomyelinase (aSMase) hydrolyze them back to ceramides, which then assemble into the mature lipid lamellae [44]. The postsecretory conversion of glucosylceramide and sphingomyelin to ceramide by β-glucocerebrosidase and aSMase, and its pH dependence, is discussed further in Section 4.4.

Ceramide catabolism proceeds through ceramidases (yielding sphingosine and FFAs), sphingosine kinases (yielding S1P), and ceramide kinase (yielding C1P), the balance between these anabolic and catabolic routes determining the steady-state ceramide profile of the epidermis [45].

### 4.2. Fatty Acid Elongation and Its Role in Ceramide Structural Diversity

**Figure 3:**
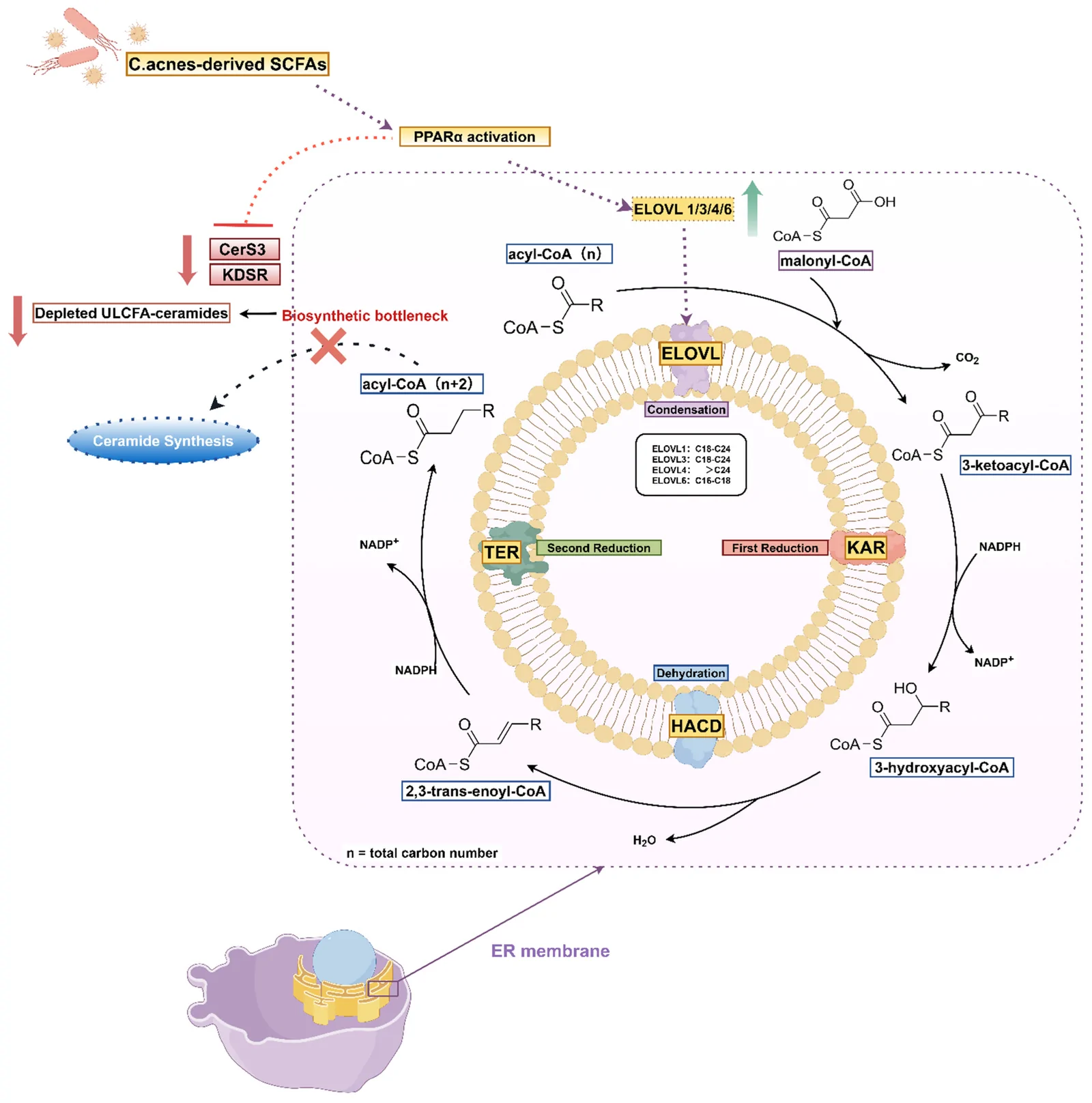
Dysregulated fatty acid elongation and biosynthetic bottleneck in acne.

The chain length of the N-acyl FA is the primary source of ceramide structural diversity and determines ceramide function in the barrier. FA elongation occurs in the ER, where fatty acyl-CoAs are extended in two-carbon increments through a rate-limiting condensation step catalyzed by the ELOVL family of elongases (ELOVL1–7) [46,47,48]. Among these, ELOVL6 catalyzes the initial elongation of palmitate (C16), whereas ELOVL4 is essential for FAs longer than 24 carbons and is therefore critical for ω-hydroxy ceramide synthesis. The elongation steps and the chain-length classes relevant to epidermal ceramide synthesis are summarized in Figure 3. For clarity, the following chain-length definitions are used throughout this review: long-chain FAs (LCFAs) = C16–C20, VLCFAs = C22–C26, and ULCFAs = ≥C28 [47]. These distinctions are functionally significant: VLCFA-containing ceramides (C22–C26) are the principal structural ceramides of the epidermal barrier (CER[NH], CER[AH], CER[NS]), while ULCFA-containing ceramides (≥C28) are the exclusive precursors of ω-hydroxy ceramides (CER[EOS], CER[EOP], CER[EOH]) that form the CLE [16,40].

### 4.3. Altered Ceramide Enzyme Expression in Acne-Prone Skin

Current research on ceramides in acne-prone skin has emphasized lipid compositional changes, while studies directly measuring the expression and activity of the biosynthetic enzymes remain limited. However, several recent investigations provide mechanistic clues.

Almoughrabie et al. demonstrated that *C. acnes* produces short-chain FAs (SCFAs) that act on normal human epidermal keratinocytes (NHEKs) through peroxisome proliferator-activated receptor α (PPARα) to stimulate lipid synthesis [7]. In NHEKs exposed to *C. acnes*-conditioned medium, the expression of ELOVL1, ELOVL3, ELOVL4, and ELOVL6 was upregulated, along with DES1, DES2, and CerS4. Concurrently, CerS3 and KDSR expression was downregulated [7]. This expression pattern, which is schematically summarized in Figure 3 as an acne-associated supply–assembly uncoupling, is mechanistically significant: ELOVL upregulation increases the supply of elongated fatty acyl-CoAs, but the concomitant downregulation of CerS3 limits the incorporation of fatty acids of C26 and longer into ceramides, while KDSR downregulation restricts the production of dihydrosphingosine, the obligate precursor for CerS3. The net effect is hypothetically consistent with the compositional finding in acne skin: a shift toward shorter-chain ceramide species at the expense of the ULCFA ω-hydroxy ceramides (CER[EOH], CER[EOS]) that are essential for barrier integrity [6]. The upregulation of CerS4 (which uses C18–C22 acyl-CoAs) may partially compensate for CerS3 loss, but the ceramides it produces are shorter and less effective at forming the highly ordered lamellar structures required for barrier function.

These results are corroborated by targeted lipidomic data from acne patients. Zhou et al. reported that fatty acid synthase (FAS) content and sphingosine levels are elevated in acne-prone skin, possibly as a compensatory response to inhibit 5α-reductase and reduce androgen-driven sebum secretion [29]. The average chain length of FAs was significantly shortened, consistent with reduced flux through the CerS3-dependent ULCFA-ceramide pathway.

This pathway, *C. acnes*→ SCFAs → PPARα → ELOVL induction + CerS3/KDSR suppression → reduced ULCFA-ceramides → barrier impairment, provides a testable working hypothesis, not an established in vivo mechanism. This hypothesis integrates current compositional, enzymatic, and functional observations. Direct validation of each step in this proposed sequence in acne-affected human skin remains to be performed.

Several questions remain. Whether the *C. acnes*-induced enzyme expression changes observed in vitro are sustained during the chronic inflammatory course of acne in vivo has not been established. The relative contribution of microbial SCFA signaling versus inflammatory cytokine signaling (Section 5) to the ceramide enzyme profile is unknown; both pathways may converge on overlapping transcriptional programs. Ceramide metabolism in the sebaceous gland, as distinct from the interfollicular epidermis, has been minimally studied.

A further consideration is the indirect effect of antibiotic therapy on ceramide metabolism. Because acne is frequently managed with long-term antibiotics (e.g., tetracyclines), and such antibiotics can remodel the skin and gut microbiome [49], it is plausible that antibiotic exposure indirectly alters ceramide metabolism through microbiome modulation. However, this relationship has not been studied and remains speculative, pending further in vivo validation.

### 4.4. Postsecretory Lipid Processing and the Acid Mantle

After secretion into the intercellular space, glucosylceramide and sphingomyelin must be converted back to ceramide by the hydrolytic enzymes β-glucocerebrosidase and acid sphingomyelinase (aSMase) before the mature lamellae can assemble, and this postsecretory processing is pH-dependent. Acne-prone skin is characterized by a more alkaline surface: Prakash et al. reported a mean skin surface pH of 6.35 ± 1.30 in 200 acne patients versus 5.09 ± 0.39 in 200 controls (*p* < 0.001) [50]. Acidic pH is required both for the multilamellar lipid assembly and for the activity of aSMase and β-glucocerebrosidase that generate barrier ceramides [51]. Rising pH impairs the activity of these two enzymes and, in parallel, the permeability barrier, while lamellar-body secretion itself is pH-independent, localizing the pH-sensitive step to postsecretory processing rather than secretion [52]. Consistent with this, acute experimental acidification of stratum corneum membrane domains restores the activity of β-glucocerebrosidase and aSMase and improves lipid processing [53]. In AD, disturbed β-glucocerebrosidase and aSMase activity is directly associated with altered stratum corneum ceramide subclass composition [54]. Although direct in vivo validation in acne is currently lacking, a similar pH-dependent enzymatic suppression may operate in the acne microenvironment. This would form a positive-feedback loop: elevated pH promotes C. acnes growth and SCFA production, which, through PPARα, suppresses CerS3 and KDSR, reducing ULCFA-ceramides and further impairing the barrier. The resulting pH increase would then reinforce the loop, so that postsecretory lipid processing and the microbial-ceramide axis mutually amplify one another. We hypothesize that a mild elevation of surface pH, in concert with *C. acnes*-derived SCFAs, suppresses ceramide-generating hydrolytic enzymes in acne-prone skin, a possibility that awaits direct testing.

## 5. Inflammatory Modulation of Ceramide Metabolism in Acne

Inflammation is already present at the microcomedone stage, before lesions become visible [55]. Beyond this local inflammatory onset, acne also carries a systemic lipid signature. Patients with acne show elevated circulating triglycerides [56]. A meta-analysis of 29 novel susceptibility loci found enrichment in Wnt and TGF-β signaling pathways [57]. This systemic signature is further supported by metabolome-wide Mendelian randomization, which links circulating lipid species such as 1-stearoylglycerol and multiple non-acylcarnitines to acne risk [58]. None of these loci converge directly on ceramide biosynthetic genes, so the cutaneous ceramide changes are better read as downstream of inflammatory and developmental signals than as effects of primary lipid gene variants [57].

Two causal directions connect inflammation and ceramide metabolism, and both appear to operate in acne. The first runs from inflammation to ceramide changes. In acne lesions, the IL-17/Th17 axis is strongly activated and drives robust expression of IL-1β, IL-6, TNF-α, and antimicrobial peptides, and this cytokine environment can suppress epidermal differentiation and lipid-processing enzymes [59,60]. Inflammation can also act through epidermal hyperproliferation, because peroxidized squalene, a hallmark of acne-associated oxidative stress, stimulates keratinocyte proliferation and activates PPARα [61]. No study has yet shown directly that this proliferation shortens ceramide chains, but the observation fits a shorter differentiation window and the reduced CER[NP] seen in psoriasis [13]. Peroxidized squalene and *C. acnes*-derived SCFAs both signal through PPARα [7,61]. This receptor therefore integrates oxidative and microbial inputs on a shared chain-length phenotype [62].

The second runs from ceramide changes to inflammation. Beyond their structural role, ceramides are precursors of bioactive mediators. In other inflammatory models, ceramide-1-phosphate (C1P) activates MAPK and PI3K–Akt signaling to promote chemokine release and leukocyte trafficking, although whether the same happens in acne-affected skin remains unclear [45]. This pro-inflammatory potential depends on chain length. Shorter species are more readily converted to C1P, whereas longer species are preferentially retained in barrier-competent lamellae [63,64]. Sphingosine-1-phosphate (S1P) is a further ceramide metabolite that signals through S1PR1–5 to add proliferative and pro-inflammatory responses [45]. The balance between ceramide, C1P, and S1P therefore shapes whether the net signal is pro-resolution or pro-inflammatory. Consistent with this, acne patients show elevated serum C16 sphingomyelin and C1P alongside reduced C24 ceramide [18]. The stratum corneum mirrors this pattern with a global reduction in protein-normalized stratum corneum ceramides and selective depletion of VLCFA species [5]. Whether these systemic changes reflect cutaneous pathology or an independent metabolic disturbance remains unresolved.

We therefore propose a bidirectional, self-reinforcing model rather than a unidirectional sequence. C1P accumulation sustains inflammation through MAPK and PI3K–Akt, while *C. acnes*-derived SCFAs suppress CerS3 and KDSR and impair barrier-competent ceramide synthesis. Each arm amplifies the other, so barrier disruption and inflammation form a feedback loop. Current cross-sectional data cannot resolve which event initiates this loop. That will require longitudinal or interventional studies.

Whether this axis is specific to acne remains to be verified. In AD, short-chain ceramide accumulation is driven instead by type 2 cytokines IL-4 and IL-13, which downregulate ELOVL3 and ELOVL6 through signal transducer and activator of transcription 6 (STAT6) [65]. This mechanism is unrelated to the acne-associated SCFA–PPARα–CerS3/KDSR pathway. Psoriasis shows a selective reduction in CER[NP] [13]. Distinct drivers thus converge on a shared chain-length phenotype, situating the *C. acnes*–SCFA pathway within the sebaceous-gland microenvironment rather than as a universal disease mechanism.

Clinically, ceramide-containing formulations improve dryness, erythema, scaling, and lesion counts in small trials [66,67]. However, a split-face trial found no significant between-group differences in TEWL, hydration, sebum, or surface pH [67]. Effective repair requires ceramide, cholesterol, and free fatty acids at a 3:1:1 molar ratio, which many commercial products may not achieve [68]. C1P and related sphingolipid mediators are emerging as candidate biomarkers, although their clinical translation requires demonstration of sensitivity, specificity, and utility for monitoring treatment response [18,44]. Targeting the CerS3–KDSR node pharmacologically faces unresolved challenges in enzyme selectivity, epidermal delivery, and off-target effects [69].

## 6. Conclusions and Future Directions

In acne vulgaris, lipidomic studies have identified reduced levels of several VLCFA- and ULCFA-ceramide subclasses, including CER[NH], CER[AH], CER[EOH], and CER[EOS], while shorter-chain species are relatively increased. At the enzymatic level, an in vitro keratinocyte study suggests that *C. acnes*-derived short-chain fatty acids signal through PPARα to suppress CerS3 and KDSR expression while upregulating ELOVL elongases. This transcriptional shift may increase the supply of elongated fatty acyl-CoAs while restricting their incorporation into ULCFA-ceramides, but this sequence has not yet been validated in vivo. Alongside these proposed enzymatic changes, the IL-17/Th17-dominated inflammatory environment coexists with elevated ceramide-1-phosphate, which can activate MAPK and PI3K–Akt pathways and amplify chemokine release. Together, the barrier defect and the inflammatory response reinforce each other. Small clinical trials have shown that ceramide-containing formulations can mitigate barrier damage and improve lesion counts when used alongside standard therapy. Whether the CerS3–KDSR node is a cause or a consequence of barrier impairment remains unresolved.

This review has limitations. The literature search was confined to English-language publications, which may introduce publication and language bias and likely underrepresents research published in Chinese and other languages. The synthesis is narrative rather than systematic. Moreover, the review synthesizes lipidomic data from both adolescent and adult cohorts, and the confounding effects of sex hormones—particularly androgens that rise during adolescence and female hormonal fluctuations—on ceramide-related enzymatic pathways have not been systematically disentangled, limiting the specificity of the conclusions to adolescent acne. Critically, the causal chain from *C. acnes*-derived SCFAs through PPARα to CerS3/KDSR suppression rests on a single in vitro keratinocyte study, and corresponding in vivo protein or mRNA expression data from acne lesions are not yet available. The mechanistic evidence rests largely on in vitro keratinocyte models, with direct enzyme activity measurements in acne-affected human skin not yet available, and the clinical trials of ceramide-containing formulations have been relatively small. Longitudinal studies tracking ceramide profiles through the natural history of individual lesions, and studies resolving whether systemic sphingolipid alterations reflect cutaneous pathology or independent metabolic disturbances, are lacking. Resolving whether the CerS3–KDSR node is a causal therapeutic target will require paired enzyme–lipidomic profiling in vivo and longitudinal studies tracking ceramide metabolism through the natural history of individual lesions.

Several specific questions merit future attention. Whether the shortened acyl chains in acne shift DES2 activity from C4-hydroxylation (phytosphingosine) toward Δ4-desaturation (sphingosine), thereby altering the CER[NP]:CER[NS] balance, has not been directly examined and merits targeted lipidomic analysis. Similarly, the postsecretory, pH-dependent contributions of β-glucocerebrosidase and acid sphingomyelinase proposed in Section 4.4 require direct measurement in acne lesions. Whether acne-associated barrier defects predispose the skin to opportunistic fungal colonization likewise awaits study.

## Figures and Tables

**Table 1:** Ceramide Subtypes Stratified by Structural Features.

	Sphingoid Base	Sphingosine (S)	Phytosphingosine (P)	6-Hydroxysphingosine (H)	Dihydrosphingosine (dS)
Fatty Acid					
**Non-hydroxy(N)**		NS	NP	NH	NdS
**α-Hydroxy(A)**		AS	AP	AH	AdS
**ω** **-Hydroxy(O)**		OS	OP	OH	OdS
**Esterifying ω-** **hydroxy (** **EO)**		EOS	EOP	EOH	EOdS

## Data Availability

No new data were created or analyzed in this study. Data sharing is not applicable to this article.

## Abbreviations

The following abbreviations are used in this manuscript:

aSMase	acid sphingomyelinase
AD	atopic dermatitis
CoA	coenzyme A
C1P	ceramide-1-phosphate
*C. acnes*	*Cutibacterium acnes*
CerS	ceramide synthase
CLE	corneocyte lipid envelope
DES	dihydroceramide desaturase
ER	endoplasmic reticulum
FA	fatty acid
FAS	fatty acid synthase
FFA	free fatty acid
IL	interleukin
KDSR	3-ketodihydrosphingosine reductase
LCFA	long-chain fatty acid
MAPK	mitogen-activated protein kinase
MCP-1	monocyte chemoattractant protein-1
NADPH	nicotinamide adenine dinucleotide phosphate
NHEK	normal human epidermal keratinocyte
PI3K–Akt	phosphoinositide 3-kinase–Akt
PNPLA1	patatin-like phospholipase domain-containing protein 1
PPARα	peroxisome proliferator-activated receptor α
S1P	sphingosine-1-phosphate
S1PR	sphingosine-1-phosphate receptor
SCFA	short-chain fatty acid
SK	sphingosine kinase
SM	sphingomyelin
SPT	serine palmitoyltransferase
STAT6	signal transducer and activator of transcription 6
TEWL	transepidermal water loss
TGF-β	transforming growth factor-β
TNF-α	tumor necrosis factor-α
ULCFA	ultra-long-chain fatty acid
VLCFA	very-long-chain fatty acid

## References

[1] Zaenglein A.L., Pathy A.L., Schlosser B.J., Alikhan A., Baldwin H.E., Berson D.S., Bowe W.P., Graber E.M., Harper J.C., Kang S. (2016). Guidelines of Care for the Management of Acne Vulgaris. J. Am. Acad. Dermatol..

[2] Williams H.C., Dellavalle R.P., Garner S. (2012). Acne Vulgaris. Lancet.

[3] Dréno B., Bagatin E., Blume-Peytavi U., Rocha M., Gollnick H. (2018). Female Type of Adult Acne: Physiological and Psychological Considerations and Management. J. Dtsch. Dermatol. Ges..

[4] Yamamoto A., Takenouchi K., Ito M. (1995). Impaired Water Barrier Function in Acne Vulgaris. Arch. Dermatol. Res..

[5] Pappas A., Kendall A.C., Brownbridge L.C., Batchvarova N., Nicolaou A. (2018). Seasonal Changes in Epidermal Ceramides Are Linked to Impaired Barrier Function in Acne Patients. Exp. Dermatol..

[6] Miner K., Murphy R., Steiss S., Burian M., Welp H., Loperfito A., O’Malley A., Misra R., Frasier K.M. (2025). Lipid Dysregulation in Sebaceous Gland Disorders and the Impact of Sphingolipid Metabolism on Acne Pathogenesis. Cureus.

[7] Almoughrabie S., Cau L., Cavagnero K., O’Neill A.M., Li F., Roso-Mares A., Mainzer C., Closs B., Kolar M.J., Williams K.J. (2023). Commensal Cutibacterium acnes Induce Epidermal Lipid Synthesis Important for Skin Barrier Function. Sci. Adv..

[8] Wertz P.W., Elias P.M., Feingold K.R. (2006). Biochemistry of Human Stratum Corneum Lipids. Skin Barrier.

[9] Michaels A.S., Chandrasekaran S.K., Shaw J.E. (1975). Drug Permeation through Human Skin: Theory and In Vitro Experimental Measurement. AIChE J..

[10] Elias P.M. (1981). Lipids and the Epidermal Permeability Barrier. Arch. Dermatol. Res..

[11] Funato K., Riezman H. (2001). Vesicular and Nonvesicular Transport of Ceramide from ER to the Golgi Apparatus in Yeast. J. Cell Biol..

[12] Masukawa Y., Narita H., Shimizu E., Kondo N., Sugai Y., Oba T., Homma R., Ishikawa J., Takagi Y., Kitahara T. (2008). Characterization of Overall Ceramide Species in Human Stratum Corneum. J. Lipid Res..

[13] Motta S., Monti M., Sesana S., Caputo R., Carelli S., Ghidoni R. (1993). Ceramide Composition of the Psoriatic Scale. Biochim. Biophys. Acta.

[14] Robson K.J., Stewart M.E., Michelsen S., Lazo N.D., Downing D.T. (1994). 6-Hydroxy-4-Sphingenine in Human Epidermal Ceramides. J. Lipid Res..

[15] Janssens M., van Smeden J., Gooris G.S., Bras W., Portale G., Caspers P.J., Vreeken R.J., Hankemeier T., Kezic S., Wolterbeek R. (2012). Increase in Short-Chain Ceramides Correlates with an Altered Lipid Organization and Decreased Barrier Function in Atopic Eczema Patients. J. Lipid Res..

[16] Ge F., Sun K., Hu Z., Dong X. (2023). Role of Omega-Hydroxy Ceramides in Epidermis: Biosynthesis, Barrier Integrity and Analyzing Method. Int. J. Mol. Sci..

[17] Ishikawa J., Narita H., Kondo N., Hotta M., Takagi Y., Masukawa Y., Kitahara T., Takema Y., Koyano S., Yamazaki S. (2010). Changes in the Ceramide Profile of Atopic Dermatitis Patients. J. Investig. Dermatol..

[18] Kaya S., Aslan İ., Kıraç E., Karaarslan T., Aslan M. (2019). Serum Sphingolipidomic Analysis in Acne Vulgaris Patients. Ann. Clin. Lab. Sci..

[19] Su Q., Hu X., Yang M., He H., Jia Y. (2024). Lipidomic Analysis of Facial Skin Surface Lipids in Acne in Young Women. Int. J. Cosmet. Sci..

[20] Cheng Y., Sun Q., Gao J., Liu Q., Tian H., Ding H., Qiao J., Chen H. (2025). Quantitative Lipidomics Profiling of Skin Surface Lipids and Skin Barrier Function Evaluation in Patients with Acne Vulgaris. Arch. Dermatol. Res..

[21] Tan Y.L., Zhang H.W., Shen J.Q., Fu X.D., Zhang Z.Y., Liu Y. (2025). Multi-Omics Analysis Reveals an Altered Skin Surface Lipid Profile in Moderate-to-Severe Acne Patients with Correlated Changes in the Skin Microbiome: A Cross-Sectional Study. Int. J. Dermatol. Venereol..

[22] Zhou M., Gan Y., He C., Chen Z., Jia Y. (2018). Lipidomics Reveals Skin Surface Lipid Abnormity in Acne in Young Men. Br. J. Dermatol..

[23] van Smeden J., Bouwstra J.A. (2016). Stratum Corneum Lipids: Their Role for the Skin Barrier Function in Healthy Subjects and Atopic Dermatitis Patients. Curr. Probl. Dermatol..

[24] Borda L.J., Wikramanayake T.C. (2015). Seborrheic Dermatitis and Dandruff: A Comprehensive Review. J. Clin. Investig. Dermatol..

[25] Miao B., Yao J., Zhang S., Guo X., Geng Y., Gong J. (2026). Public Health Risks of Pathogenic Fungi in China. iFungi.

[26] Knox S., O’Boyle N.M. (2021). Skin Lipids in Health and Disease: A Review. Chem. Phys. Lipids.

[27] Nicollier M., Massengo T., Rémy-Martin J.P., Laurent R., Adessi G.-L. (1986). Free Fatty Acids and Fatty Acids of Triacylglycerols in Normal and Hyperkeratotic Human Stratum Corneum. J. Investig. Dermatol..

[28] Miller S.J., Aly R., Shinefeld H.R., Elias P.M. (1988). In Vitro and In Vivo Antistaphylococcal Activity of Human Stratum Corneum Lipids. Arch. Dermatol..

[29] Zhou M., Yang M., Zheng Y., Dong K., Song L., He C., Liu W., Wang Y., Jia Y. (2020). Skin Surface Lipidomics Revealed the Correlation between Lipidomic Profile and Grade in Adolescent Acne. J. Cosmet. Dermatol..

[30] Wertz P.W., Miethke M.C., Long S.A., Strauss J.S., Downing D.T. (1985). The Composition of the Ceramides from Human Stratum Corneum and from Comedones. J. Investig. Dermatol..

[31] Letawe C., Boone M., Piérard G.E. (1998). Digital Image Analysis of the Effect of Topically Applied Linoleic Acid on Acne Microcomedones. Clin. Exp. Dermatol..

[32] Kao J.S., Garg A., Mao-Qiang M., Crumrine D., Ghadially R., Feingold K.R., Elias P.M. (2001). Testosterone Perturbs Epidermal Permeability Barrier Homeostasis. J. Investig. Dermatol..

[33] Denda M., Koyama J., Hori J., Horii I., Takahashi M., Hara M., Tagami H. (1993). Age- and Sex-Dependent Change in Stratum Corneum Sphingolipids. Arch. Dermatol. Res..

[34] Makrantonaki E., Zouboulis C.C. (2007). Testosterone Metabolism to 5α-Dihydrotestosterone and Synthesis of Sebaceous Lipids Is Regulated by the Peroxisome Proliferator-Activated Receptor Ligand Linoleic Acid in Human Sebocytes. Br. J. Dermatol..

[35] Melnik B., Kinner T., Plewig G. (1988). Influence of Oral Isotretinoin Treatment on the Composition of Comedonal Lipids: Implications for Comedogenesis in Acne Vulgaris. Arch. Dermatol. Res..

[36] Hanada K. (2003). Serine Palmitoyltransferase, a Key Enzyme of Sphingolipid Metabolism. Biochim. Biophys. Acta.

[37] Kihara A., Igarashi Y. (2004). FVT-1 Is a Mammalian 3-Ketodihydrosphingosine Reductase with an Active Site That Faces the Cytosolic Side of the Endoplasmic Reticulum Membrane. J. Biol. Chem..

[38] Michel C., van Echten-Deckert G., Rother J., Sandhoff K., Wang E., Merrill A.H. (1997). Characterization of Ceramide Synthesis: A Dihydroceramide Desaturase Introduces the 4,5-Trans-Double Bond of Sphingosine at the Level of Dihydroceramide. J. Biol. Chem..

[39] Rabionet M., Gorgas K., Sandhoff R. (2014). Ceramide Synthesis in the Epidermis. Biochim. Biophys. Acta.

[40] Jennemann R., Rabionet M., Gorgas K., Epstein S., Dalpke A., Rothermel U., Bayerle A., van der Hoeven F., Imgrund S., Kirsch J. (2012). Loss of Ceramide Synthase 3 Causes Lethal Skin Barrier Disruption. Hum. Mol. Genet..

[41] Radner F.P.W., Marrakchi S., Kirchmeier P., Kim G.J., Ribierre F., Kamoun B., Abid L., Leipoldt M., Turki H., Schempp W. (2013). Mutations in CERS3 Cause Autosomal Recessive Congenital Ichthyosis in Humans. PLoS Genet..

[42] Ohno Y., Kamiyama N., Nakamichi S., Kihara A. (2017). PNPLA1 Is a Transacylase Essential for the Generation of the Skin Barrier Lipid ω-O-Acylceramide. Nat. Commun..

[43] Ternes P., Franke S., Zähringer U., Sperling P., Heinz E. (2002). Identification and Characterization of a Sphingolipid Δ4-Desaturase Family. J. Biol. Chem..

[44] Jensen J.M., Förl M., Winoto-Morbach S., Seite S., Schunck M., Proksch E., Schütze S. (2005). Acid and Neutral Sphingomyelinase, Ceramide Synthase, and Acid Ceramidase Activities in Cutaneous Aging. Exp. Dermatol..

[45] Gomez-Muñoz A., Presa N., Gomez-Larrauri A., Rivera I.-G., Trueba M., Ordoñez M. (2016). Control of Inflammatory Responses by Ceramide, Sphingosine 1-Phosphate and Ceramide 1-Phosphate. Prog. Lipid Res..

[46] Jakobsson A., Westerberg R., Jacobsson A. (2006). Fatty Acid Elongases in Mammals: Their Regulation and Roles in Metabolism. Prog. Lipid Res..

[47] Tanno H., Sassa T., Sawai M., Kihara A. (2021). Production of Branched-Chain Very-Long-Chain Fatty Acids by Fatty Acid Elongases and Their Tissue Distribution in Mammals. Biochim. Biophys. Acta-Mol. Cell Biol. Lipids.

[48] Sassa T., Kihara A. (2014). Metabolism of Very Long-Chain Fatty Acids: Genes and Pathophysiology. Biomol. Ther..

[49] Chien A.L., Tsai J., Leung S., Mongodin E.F., Nelson A.M., Kang S., Garza L.A. (2019). Association of Systemic Antibiotic Treatment of Acne with Skin Microbiota Characteristics. JAMA Dermatol..

[50] Prakash C., Bhargava P., Tiwari S., Majumdar B., Bhargava R.K. (2017). Skin Surface pH in Acne Vulgaris: Insights from an Observational Study and Review of the Literature. J. Clin. Aesthet. Dermatol..

[51] Brooks S.G., Mahmoud R.H., Lin R.R., Fluhr J.W., Yosipovitch G. (2025). The Skin Acid Mantle: An Update on Skin pH. J. Investig. Dermatol..

[52] Elias P.M. (2015). Stratum Corneum Acidification: How and Why?. Exp. Dermatol..

[53] Hachem J.P., Roelandt T., Schürer N., Pu X., Fluhr J., Giddelo C., Man M.Q., Crumrine D., Roseeuw D., Feingold K.R. (2010). Acute Acidification of Stratum Corneum Membrane Domains Using Polyhydroxyl Acids Improves Lipid Processing and Inhibits Degradation of Corneodesmosomes. J. Investig. Dermatol..

[54] Boer D.E., van Smeden J., Al-Khakany H., Melnik E., van Dijk R., Absalah S., Vreeken R.J., Haenen C.C., Lavrijsen A.P., Overkleeft H.S. (2020). Skin of Atopic Dermatitis Patients Shows Disturbed β-Glucocerebrosidase and Acid Sphingomyelinase Activity That Relates to Changes in Stratum Corneum Lipid Composition. Biochim. Biophys. Acta-Mol. Cell Biol. Lipids.

[55] Jeremy A.H.T., Holland D.B., Roberts S.G., Thomson K.F., Cunliffe W.J. (2003). Inflammatory Events Are Involved in Acne Lesion Initiation. J. Investig. Dermatol..

[56] Sobhan M., Seif Rabiei M.A., Amerifar M. (2020). Correlation Between Lipid Profile and Acne Vulgaris. Clin. Cosmet. Investig. Dermatol..

[57] Mitchell B.L., Saklatvala J.R., Dand N., Hagenbeek F.A., Li X., Min J.L., Thomas L., Bartels M., Hottenga J.J., Lupton M.K. (2022). Genome-Wide Association Meta-Analysis Identifies 29 New Acne Susceptibility Loci. Nat. Commun..

[58] Li M., Zhan D.D., Fan L.L., Wang Y., Hu X.H., Zhang M., Zhou Z. (2025). Unraveling the Causal Relationship Between Blood Metabolites and Acne: A Metabolomic Mendelian Randomization Study. J. Cosmet. Dermatol..

[59] Kelhälä H.L., Palatsi R., Fyhrquist N., Lehtimäki S., Väyrynen J.P., Kallioinen M., Kubin M.E., Greco D., Tasanen K., Alenius H. (2014). IL-17/Th17 Pathway Is Activated in Acne Lesions. PLoS ONE.

[60] Furue M., Furue K., Tsuji G., Nakahara T. (2020). Interleukin-17A and Keratinocytes in Psoriasis. Int. J. Mol. Sci..

[61] Ottaviani M., Alestas T., Flori E., Mastrofrancesco A., Zouboulis C.C., Picardo M. (2006). Peroxidated Squalene Induces the Production of Inflammatory Mediators in HaCaT Keratinocytes: A Possible Role in Acne Vulgaris. J. Investig. Dermatol..

[62] Condrò G., Sciortino R., Perugini P. (2023). Squalene Peroxidation and Biophysical Parameters in Acne-Prone Skin: A Pilot “In Vivo” Study. Pharmaceuticals.

[63] Grösch S., Schiffmann S., Geisslinger G. (2012). Chain Length-Specific Properties of Ceramides. Prog. Lipid Res..

[64] Hannun Y.A., Obeid L.M. (2018). Sphingolipids and Their Metabolism in Physiology and Disease. Nat. Rev. Mol. Cell Biol..

[65] Berdyshev E., Goleva E., Bronova I., Dyjack N., Rios C., Jung J., Taylor P., Jeong M., Hall C.F., Richers B.N. (2018). Lipid Abnormalities in Atopic Skin Are Driven by Type 2 Cytokines. JCI Insight.

[66] Draelos Z.D., Baalbaki N., Colon G., Dréno B. (2023). Ceramide-Containing Adjunctive Skin Care for Skin Barrier Restoration During Acne Vulgaris Treatment. J. Drugs Dermatol..

[67] Tempark T., Shem A., Lueangarun S. (2024). Efficacy of Ceramides and Niacinamide-Containing Moisturizer versus Hydrophilic Cream in Combination with Topical Anti-Acne Treatment in Mild to Moderate Acne Vulgaris: A Split Face, Double-Blinded, Randomized Controlled Trial. J. Cosmet. Dermatol..

[68] Man M.Q., Feingold K.R., Thornfeldt C.R., Elias P.M. (1996). Optimization of Physiological Lipid Mixtures for Barrier Repair. J. Investig. Dermatol..

[69] You Z., Zhuang K., Sugita T., Ran Y. (2026). Novel Strategies Against Fungal Infections: Repurposing Existing Drugs and Advancing Nanotherapeutics. iFungi.

